# Cortico-cortical stimulation and robot-assisted therapy (CCS and RAT) for upper limb recovery after stroke: study protocol for a randomised controlled trial

**DOI:** 10.1186/s13063-023-07849-1

**Published:** 2023-12-21

**Authors:** Alex Martino Cinnera, Sonia Bonnì, Alessia D’Acunto, Michele Maiella, Matteo Ferraresi, Elias Paolo Casula, Valentina Pezzopane, Marco Tramontano, Marco Iosa, Stefano Paolucci, Giovanni Morone, Giuseppe Vannozzi, Giacomo Koch

**Affiliations:** 1 Scientific Institute for Research, Hospitalization and Health Care IRCCS Santa Lucia Foundation, Rome, Italy; 2 Department of Movement, Human and Health Sciences, University of Rome “Foro Italico”, Rome, Italy; 3 Department of System Medicine, University of Tor Vergata, Rome, Italy; 4 Department of Biomedical and Neuromotor Sciences (DIBINEM), Alma Mater University of Bologna, Bologna, Italy; 5 Unit of Occupational Medicine, IRCCS Azienda Ospedaliero-Universitaria di Bologna, Bologna, Italy; 6 Department of Psychology, Sapienza University of Rome, 00185 Rome, Italy; 7 Department of Life, Health and Environmental Sciences, University of L’Aquila, 67100 L’Aquila, Italy; 8 Department of Neuroscience and Rehabilitation, University of Ferrara, Ferrara, Italy

**Keywords:** Transcranial magnetic stimulation, Paired associative stimulation, Brain stimulation, Clinical trial, Neuroplasticity, Neurorehabilitation, Recovery, Arm, Therapy

## Abstract

**Background:**

Since birth, during the exploration of the environment to interact with objects, we exploit both the motor and sensory components of the upper limb (UL). This ability to integrate sensory and motor information is often compromised following a stroke. However, to date, rehabilitation protocols are focused primarily on recovery of motor function through physical therapies. Therefore, we have planned a clinical trial to investigate the effect on functionality of UL after a sensorimotor transcranial stimulation (real vs sham) in add-on to robot-assisted therapy in the stroke population.

**Methods:**

A randomised double-blind controlled trial design involving 32 patients with a single chronic stroke (onset > 180 days) was planned. Each patient will undergo 15 consecutive sessions (5 days for 3 weeks) of paired associative stimulation (PAS) coupled with UL robot-assisted therapy. PAS stimulation will be administered using a bifocal transcranial magnetic stimulator (TMS) on the posterior-parietal cortex and the primary motor area (real or sham) of the lesioned hemisphere. Clinical, kinematics and neurophysiological changes will be evaluated at the end of protocol and at 1-month follow-up and compared with baseline. The Fugl-Meyer assessment scale will be the primary outcome. Secondly, kinematic variables will be recorded during the box-and-block test and reaching tasks using video analysis and inertial sensors. Single pulse TMS and electroencephalography will be used to investigate the changes in local cortical reactivity and in the interconnected areas.

**Discussion:**

The presented trial shall evaluate with a multimodal approach the effects of sensorimotor network stimulation applied before a robot-assisted therapy training on functional recovery of the upper extremity after stroke. The combination of neuromodulation and robot-assisted therapy can promote an increase of cortical plasticity of sensorimotor areas followed by a clinical benefit in the motor function of the upper limb.

**Trial registration:**

ClinicalTrials.gov NCT05478434. Registered on 28 Jul 2022.

## Administrative information

Note: the numbers in curly brackets in this protocol refer to SPIRIT checklist item numbers. The order of the items has been modified to group similar items (see http://www.equator-network.org/reporting-guidelines/spirit-2013-statement-defining-standard-protocol-items-for-clinical-trials/).

**Table:** 

Title {1}	Cortico-Cortical Stimulation and Robot-Assisted Therapy (CCS&RAT) for upper limb recovery after stroke: study protocol for a randomised controlled trial
Trial registration {2a and 2b}.	NIH U.S. National Library of Medicine; ClinicalTrials.gov (NCT05478434); Registered Jul 28, 2022. The register used for registration does not meet all items from the World Health Organization Trial Registration Data Set.
Protocol version {3}	Protocol version 1.3 of the 2021/04/09.
Funding {4}	The Santa Lucia Foundation supplies the equipment and finances the consumables. PhD funding from AMC, 10% of the amount of the scholarship for research activity in accordance with law no. 226 of 14 December 2021 of Italian Ministry of University and Research, are partially devolved in the current study. This research has not received other financial support from any funding agency in the public, commercial, or not-for-profit sectors.
Author details {5a}	^1^Scientific Institute for Research, Hospitalization and Health Care IRCCS Santa Lucia Foundation, Rome, Italy. ^2^Department of Movement, Human and Health Sciences, University of Rome "Foro Italico", Rome, Italy. ^3^Department of Psychology, Sapienza University of Rome, 00185 Rome, Italy. ^4^Department of Life, Health and Environmental Sciences, University of L'Aquila, 67,100 L'Aquila, Italy. ^5^Department of Neuroscience and Rehabilitation, University of Ferrara, Italy.
Name and contact information for the trial sponsor {5b}	IRCCS Santa Lucia Foundation Scientific Institute for Research, Hospitalization and Health Care Ardeatina street 306–354, 00179, Rome, Italy tel. number + 39 06.5150.11 Fax + 39 06.5032.097 info@hsantalucia.it
Role of sponsor {5c}	The sponsor is responsible for ensuring approvals are obtained prior to starting the trial. The sponsor has no role in the study design, data collection or publication.

## Introduction

### Background and rationale {6a}

Up to 80% of stroke survivors have upper limb (UL) impairments early after stroke and a few of these demonstrate complete functional recovery after 6 months from the stroke event [1]. Impairment of the UL is one of the factors that contribute to reducing the overall quality of life impacting significantly social participation and the odds of return to professional activities [2]. The impairment of the UL is due to motor and sensory alteration that could compromise the sensorimotor integration, which is a complex process in the central nervous system which produces task-specific motor output based on selective and rapid integration of sensory information from multiple sources [3]. Specifically, sensory and motor signals are integrated by specific brain circuits during goal-directed behaviour and active exploration of the sensory environment [4]. The posterior parietal cortex (PPC) is a potential circuit where sensorimotor integration could occur during an active somatosensorial task [4]. Indeed, PPC is a site of massive confluence of visual, tactile, proprioceptive and vestibular signals [5]. This area may be involved in transforming information about the location of targets in space, into signals related to motor intentions [6]. This process likely occurs through parietal-motor connections, which are known to be involved in the transfer of relevant sensitive information for planning, reaching and grasping. Sensorimotor integration can be explained by anatomical cortico-cortical connections between PPC and the primary motor area (M1) by bundles of the superior longitudinal fasciculus [7]. Indeed, these mechanisms are not fixed but susceptible to rapid adaptations and modulation, through Hebbian-like plasticity mechanisms [8], in different populations of postsynaptic neurons. Recently, it has been found that paired associative stimulation (PAS) of PPC and M1, by means of bi-focal TMS, can modulate M1 excitability [9]. This information reinforces the hypothesis that modulation of PPC-M1 connectivity can be used as a new approach to modify motor excitability and sensorimotor interaction [8]. Moreover, Hebbian plasticity is a key factor of learning-dependent mechanisms for recovery of the UL after stroke [10].

Parallel, in the past decades, robotic therapy often focuses on increased strength and joint movement. Robot-assisted training can induce a plastic reorganisation at the muscular afferents, spinal motor neurons, interneuron system and beyond and facilitates neural plasticity and motor relearning through goal-oriented training programme. This technology can assist patients in their movements and of generating a biomechanical biofeedback based on measurements of movement. This biofeedback can sometimes be referred to as augmented feedback, providing the user with additional information, above and beyond the information that is naturally available to them as opposed to the sensory (or intrinsic) feedback [11]. The augmented feedback has greater clinical effects than sensory feedback and generates a facilitation in the neural plasticity after a brain injury. The robotics device allows to train patients in an intensive, task-oriented and top-down therapy way, increasing patients’ compliance and motivation. The cognitive top-down stimulation is allowed by the introduction of visual feedback performed through exergaming [12]. In addition, by using computer-assisted devices for regaining UL function, the robot can easily apply new constraints, to optimise the required movement pattern. Specifically, the robotic exoskeleton can assist the paretic UL in a large 1D, 2D or 3D environment by promoting movement [13]. Therefore, the complexity of a motor task can be controlled for more precisely with robotics than in conventional treatment approaches. Recently, the development of new intervention strategies has been proposed combining neurostimulation of a target brain area with neurorehabilitation, such as physical therapy or virtual reality [14]. Although both TMS and robot-assisted therapy (RAT) have shown individually promising effects in UL recovery after a stroke [15–17], their combination has not been tested to date. In detail, a paired stimulation of two interconnected areas of the cerebral cortex has not been combined with a specific and precisely controlled rehabilitation through robotic exoskeleton. The use of robot-assisted training may also drastically reduce the bias induced by neurorehabilitation administered by different physiotherapists, which intrinsically can undergo greater discrepancies between exercises. The possibility of a robot to measure body movement, through its embodied motion sensors, allows for the recording of data related to UL performance, also providing monitoring of motor training during the trials.

## Objectives {7}

We expect that the neurostimulation of the PPC-M1 network combined with robot-assisted training (experimental group) may be more effective than sham stimulation (control group). The assumption is that the PAS promotes a direct modulation of cortical plasticity while the robot-assisted exergaming provides only an indirect stimulation of circuits involved in grasp and reaching movements.

## Trial design {8}

A randomised, parallel groups, double-blind, two-arms, sham-controlled trial will be implemented to investigate the effects of the PPC-M1 PAS in add-on to RAT. Neurologists, PM&R, engineers, psychologists and physiotherapists were involved in the study design. No patient or public participation was foreseen.

## Methods: participants, interventions and outcomes

### Study setting {9}

All procedures will be performed in the clinical and behavioural neurology department and in the cognitive, motor and neuroimaging neurorehabilitation department of the Santa Lucia Foundation Hospital. Specifically, the brain stimulation will be administered in the Non-Invasive Brain Stimulation Unit (NIBSU) and the RAT in the rehabilitation unit 6.

### Eligibility criteria {10}

**Fig. 1:**
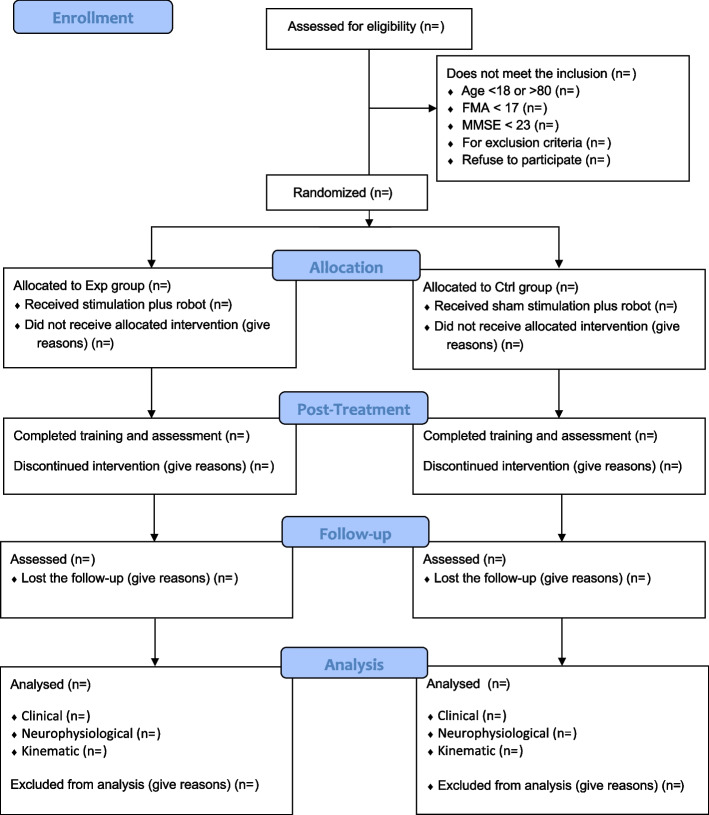
Inclusion flowchart

All patients with single ischemic stroke in the area of the middle cerebral artery in the chronic phase (> 180 days to stroke) that reported a severe-to-mild UL hemiparesis (Fugl-Meyer scale score < 52) will be screened for inclusion (Fig. 1). Inclusion criteria were as follows: (1) first ever chronic ischemic stroke; (2) hemiparesis due to left or right subcortical or cortical lesion in the territory of the middle cerebral artery; and (3) residual UL impairment (FMA-UE < 52). Exclusion criteria: (1) patients older than 80 years; (2) history of seizures; (3) severe general impairment or concomitant diseases (e.g. Parkinson’s disease, multiple sclerosis); (4) treatment with benzodiazepines, baclofen, antidepressants and botulinum toxin; (5) intracranial metal implants; (6) cardiac pacemaker; (7) pregnancy status; (8) orthopaedic contraindications for UL (e.g. shoulder periarthritis, Dupuytren’s disease); (9) UL pain; (10) cognitive impairment (MMSE score < 23); (11) and presence of unilateral spatial neglect (evaluated through a clinical and functional assessment).

### Who will take informed consent? {26a}

Before starting the trial, the principal investigator or a sub-investigator will explain the experimental procedure and collect a written informed consent signed from each participant.

### Additional consent provisions for collection and use of participant data and biological specimens {26b}

In the consent form, participants will be requested to allow the utilisation of their data in accordance with local privacy policy. In the event of drop-out, they will be requested to indicate willingness to allow the utilisation of acquired data. Additionally, participants will be asked for their consent to share pertinent data with the sponsors. This trial does not involve the collection and storage of biological samples.

### Interventions

#### Explanation for the choice of comparators {6b}

We choose a combined placebo stimulation plus active-control robot-assisted comparator to reduce the bias of perceived allocation. Specifically, both groups will receive, real (Experimental group—Exp) or sham (Control group (Ctrl)), cortico-cortical PAS immediately before each session of RAT training. The Ctrl group received the same RAT protocol of the Exp group. Each participant will undergo 15 consecutive sessions (5 times a week, for 3 weeks) of PPC-M1 PAS (real or sham) in add-on to 30 min of RAT in a supervised clinical setting.

#### Intervention description {11a}

##### Transcranial magnetic stimulation procedure

A bifocal TMS will be applied to repeatedly activate the connection between the PPC and the M1 of the lesioned hemisphere [6]. Paired-pulse stimulation protocol, with 5-ms interstimulus interval between the two pulses, will be done through two Magstim 200 stimulators connected in a bi-stim mode. To stimulate the PPC area (90% rMT), the centre of the coil will be positioned over P4 (10–20 EEG system) tangentially to the skull with the handle pointing downward and slightly medial (15°) to induce a posterior anterior-directed current in the underlying cortical tissue. To stimulate the M1 area (120% rMT), the coil will be placed tangentially to the scalp at a 45° angle to the midline to induce a posterior-anterior current flow across the central sulcus. Differently from the Exp group, the Ctrl group will receive sham stimulation with a coil inclination of 90° with respect to the scalp.

##### Robot-assisted therapy procedure

**Fig. 2:**
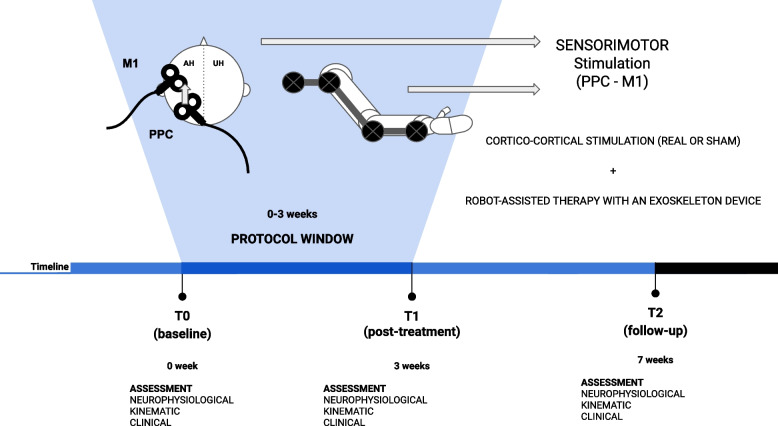
Cortico-cortical stimulation and robot-assisted training protocol

The RAT training will consist of 30-min exercises miming reaching and grasping movements of the UL. To perform the training, it will use the Armeo® Power II (Hocoma), an integrative system composed of a robotic exoskeleton device connected to a laptop for audio-visual biofeedback. The robotic exoskeleton, used for the therapy, is composed of an orthosis for the UL with six degrees of freedom: three for the shoulder, one for the elbow flexion, one for the forearm supination and one for the wrist flexion. Each joint is powered by a motor and equipped with two angle sensors. The device can support the patient’s UL weight, providing a feeling of fluctuation. The interface used for the exergame is designed to simulate UL gestures, providing a simple virtual environment [16]. All RAT sessions will be supervised by a specialised physical therapist with experience in the field of robotic rehabilitation (Fig. 2).

#### Criteria for discontinuing or modifying allocated interventions {11b}

There will be no special criteria for discontinuing or modifying allocated interventions.

#### Strategies to improve adherence to interventions {11c}

In the present study, all sessions will be administered by a study investigator. All sessions will be recorded and reported in an adherence note.

#### Relevant concomitant care permitted or prohibited during the trial {11d}

Participants should continue to take medications for other conditions as normal. However, if it is anticipated that the participant will need benzodiazepines, baclofen and antidepressants during the intervention phase, they will be ineligible for entry into the study. If a patient needed a treatment with botulinum toxin injection for upper limb spasticity, the treatment can be performed 4 months before trial or at the end of the study period (after follow-up) worth exclusion from the study.

#### Provisions for post-trial care {30}

There are no provisions for post-trial care given the anticipated low risk of harm from a participant’s involvement in this trial [18, 19]. At the end of the trial, participants will return to the standard care according to the medical indication and current guidelines.

### Outcomes {12}

The primary outcome of the study is the difference between the Exp group and Ctrl group in functional mobility of the UL. It will be measured using the Fugl-Meyer Assessment Scale for Upper Extremity (FMA-UE).

Secondly, we will investigate clinical, kinematic and neurophysiological changes between the two groups using:

- The Box and Block Test to assess manual dexterity.
- The Modified Ashworth Scale to assess spasticity.
- Kinematics variables, recorded via inertial measurement units and motion analysis during three-reaching tasks and the Box-and-Block test, to assess functional movements.
- TMS-evoked potentials, recorded via TMS-EEG, to assess cortical reactivity.
- TRSP, recorded via TMS-EEG, to assess oscillatory dynamics of the PPC and M1.
- PLV, recorded via TMS-EEG, to assess the actual connectivity between a targeted area with respect to the entire neural network.

### Participant timeline {13}

**Fig. 3:**
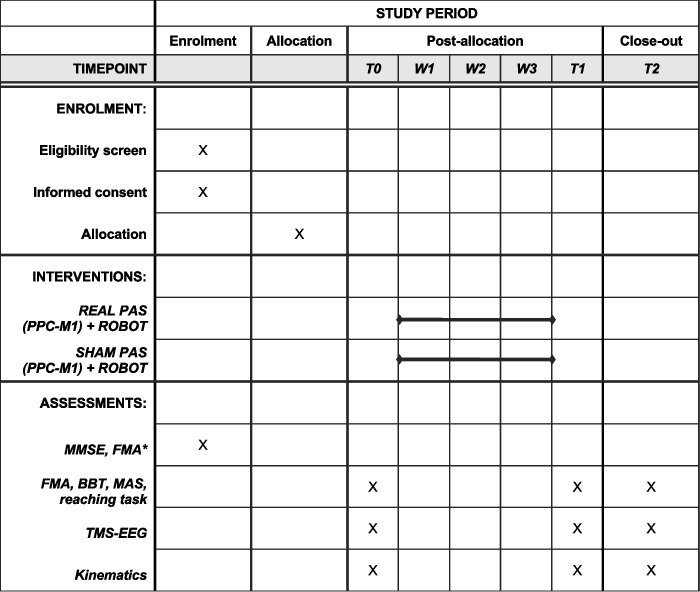
Complete treatment and assessment phases

After screening, all patients will be tested for motor (FMA) and cognitive function (MMSE). If eligible for the study, their evaluation will be completed in 72 h before they start the experimental treatment. After evaluation, each patient will perform the 3 weeks of treatment. During the 72 h, after the last session, we will perform the post-treatment evaluation. The follow-up evaluation will be performed 28 days after the end of the treatment with a 3-day margin of tolerance. The complete treatment and assessment phases are available in Fig. 3.

### Sample size {14}

According to an a priori Power analysis, considering 80% power and a 95% CI, 32 patients would be needed for necessary sample size, based on previously published work on cortical plastic changes induced by neurostimulation via TMS [14].

### Recruitment {15}

We will screen subjects admitted to the hospital until the target population is achieved (32 subjects). In case of heterogeneity of demographic characteristics between the two groups of study, the screening may continue after the anticipated sample size.

### Assignment of interventions: allocation

#### Sequence generation {16a}

A random allocation software for parallel group randomisation will be used to generate a consecutive allocation list.

#### Concealment mechanism {16b}

Allocation concealment will be ensured, the randomisation code will not be generated until the patient has completed all baseline measurements.

#### Implementation {16c}

After the baseline evaluation, the PI or Co-PI requests the allocation to the designated sub-investigator (not involved in the assessment and treatment). The allocation will be revealed exclusively to the neurophysiology technician who will be in charge of performing the stimulation protocol.

### Assignment of interventions: blinding

#### Who will be blinded {17a}

Assessments will be conducted by two assessors blind to treatment allocation. Patients and their caregivers will be instructed during recruitment about the impossibility to receive information about the allocation, even when they explicitly request it.

#### Procedure for unblinding if needed {17b}

Unblinding can be requested and managed by PI only for exceptional circumstances when knowledge of the actual treatment is essential for further management of the patient. In this case, the PI will provide communication with the Clinical Trial Center (CTC) and will include this information in the disseminations.

### Data collection and management

#### Plans for assessment and collection of outcomes {18a}

Clinical, instrumental and neurophysiological evaluation will be assessed at baseline (T0); after the 3 weeks of treatment (T1); and after 7 weeks from the baseline as a follow-up—4 weeks from the end of the treatment—(T2).

Demographics data (i.e. age, gender, lesion site) will be acquired through clinical documentation provided during the screening procedure. All collected data will be stored in an online data sheet protected with a password. To promote data quality, all raters were specifically trained in the administration of the clinical scales and in kinematics evaluation.

##### Clinical and kinematic assessment

**Fig. 4:**
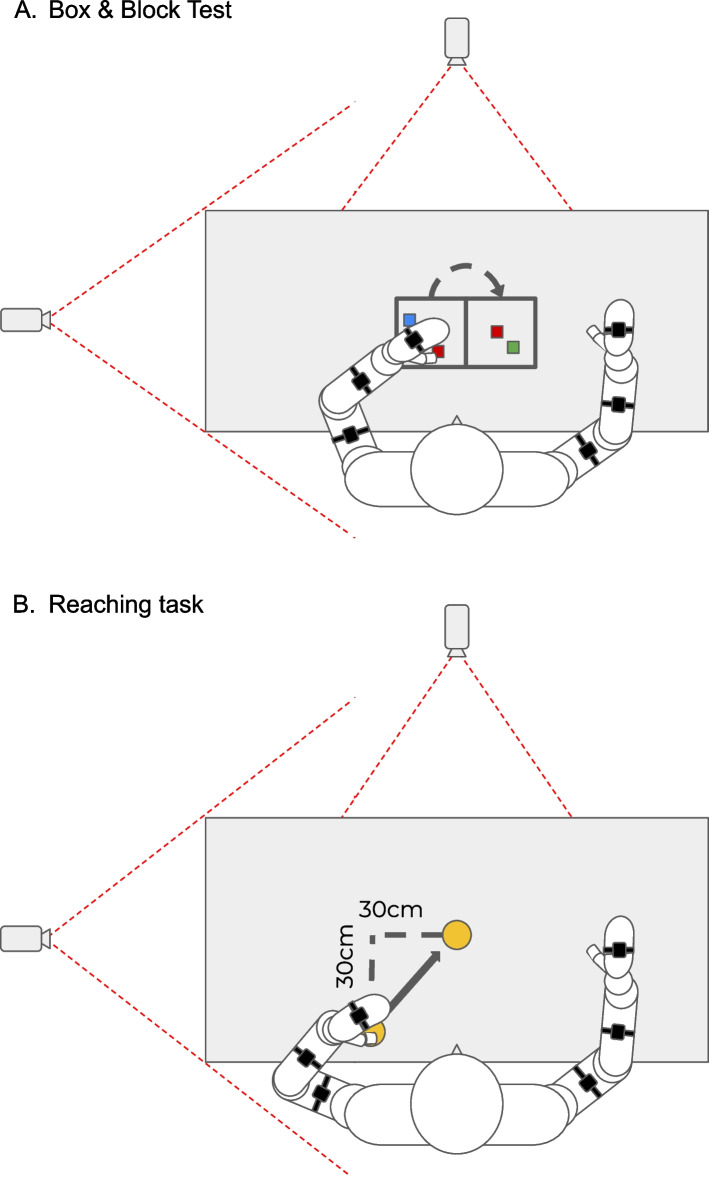
**A** Box and Block Test. **B** Reaching task

A battery of scales and functional tests will be performed to assess functionality of UL as follows: (*i*) the Fugl-Meyer Assessment scale for upper extremity (FMA-UE) for motor-sensory pain and range of motion functions of UL such as primary outcome; (*ii*) the Box and Block test (BBT) for functional mobility (Reaching and grasp/grasp-relax); (*iii*) the Modified Ashworth scale (MAS) for muscle tone function; (*iv*) kinematic assessment of reaching tasks. The FMA-UE is a comprehensive measurement tool for motor function after stroke that was shown to be valid, reliable and responsive to change. The FMA-UE showed excellent inter-rater reliability (ICC of 0.99) and test–retest reliability (ICC = 0.97 for motor score; ICC = 0.81 for sensation, ICC = 0.95 for passive joint motion/pain) [20]. Among other FMA-UE domains, the motor domain is the most widely used, having the primary value of monitoring motor recovery after stroke. Most items in the UL motor domain are based on patient motion, although reflex or resistance must be measured in a few items [21]. The BBT is an instrumented measure of gross manual dexterity, performed by counting the number of blocks that are moved from one compartment of a box to another compartment within 60 s (after 15 s of trial) (Fig. 4A). BBT showed an excellent inter-rater reliability (*r* = 1.00; *r* = 0.99) and test–retest reliability (ICC = 0.97; ICC = 0.96) for the right and left hand, respectively [22, 23]. The MAS is a 6-point clinical measure of muscle spasticity, widely used in neurological patients. MAS showed a good intra-rater reliability (weighted kappa = 0.83) and inter-rater reliability (Kendall’s tau-b = 0.84) [24, 25]. About the reaching task, the patient is sitting in front of a table with the hand on an initial predefined target. The patient is then instructed to reach from the rest position, for three times, a second target signed 30 cm ahead and 30 cm to the midline (Fig. 4B). Both hands will be tested.

UL linear 3D acceleration and angular velocity will be recorded using inertial measurement units (IMUs). IMU measures are provided with respect to a sensor-embedded frame, generally coinciding with the geometrical axes of the case in which the IMU is housed [26]. This technology can be exploited to obtain UL range of motion and further kinematic features (i.e. functional mobility and activity of daily living tasks) while the patient moves the UL freely [27]. Video analysis is performed by three video cameras placed frontally and sideways (bilaterally) to the subject with an angle of 90°, and then, motion tracking of moving targets will be performed. Both BBT and reaching tasks will be performed with both ULs starting from the unaffected side.

##### Neurophysiological assessment

We will use 120 single pulses for each area (M1 and PPC bilaterally) during electroencephalographic recordings (EEG) to measure changes in the local activity of the stimulated area and on its cortico-cortical connections. The combined use of TMS-EEG represents one of the most promising approaches in the investigation of brain dynamics, in terms of cortical activity and connectivity. Indeed, EEG can record the postsynaptic potentials generated by the TMS-evoked neuronal depolarisation providing direct information on the neurophysiological state of the stimulated area and on its connections over the cortex. Recently, the main components of TEPs that can be detected in the first 200 ms after the probing TMS pulse have been put in relation with the activity of GABAergic interneurons [28].

#### Plans to promote participant retention and complete follow-up {18b}

To promote retention, we planned a short-time follow-up (4 weeks) to maximise the completeness of data collection. In this way, we hypothesise a low number of non-retention cases (< 20%).

#### Data management {19}

All data are collected electronically through an interface that complies with European (GDPR No. 679/2016) and Italian (D.L. 101/2018) data protection guidelines. Personal data and contacts will be recorded in a separate dataset and identified via an alpha-numeric ID.

#### Confidentiality {27}

To ensure privacy, an alpha-numeric ID for each patient will be assigned to preserve personal information and contacts.

#### Plans for collection, laboratory evaluation and storage of biological specimens for genetic or molecular analysis in this trial/future use {33}

No biological specimens will be collected in the present study.

### Statistical methods

#### Statistical methods for primary and secondary outcomes {20a}

Data characterised by normal distribution will be expressed as means with standard deviations. Parameters not normally distributed will be expressed as medians with the interquartile ranges. Parametric data will be analysed with a 3 × 2 mixed model ANOVA considering time (TIME: T0 vs T1 vs T2) and group allocation (GROUP: Real vs Ctrl) as within and between factors, respectively, with *α* = 0.05. Each significant interaction (TIME × GROUP) will be further explored with the Bonferroni post hoc correction. Nonparametric data will be investigated with Friedman’s test and, if *p* < 0.05, with Wilcoxon test such as post hoc. Correlations (Pearson for normal distributed data or Spearman for non-normal distributed data) and regression analysis will be performed between clinical, kinematic and neurophysiological data to investigate casual and causal interaction respectively. Sub-group analysis can be performed after severity stratification (i.e. FMA < 17).

#### Interim analyses {21b}

Interim analysis will be performed only once when the outcomes of 20 patients will be made available. The sub-investigator designated to manage randomisation will perform the analysis according to the methodology described in the statistical planning. The results will be discussed with the PI. In case of differences in demographic and clinical characteristics between the two groups, the further recruitment will follow a pseudo-randomisation taking into account the balance in the following factors: age, sex, lesion side, stroke onset and UL severity.

#### Methods for additional analyses (e.g. subgroup analyses) {20b}

Sub-group analyses are not foreseen.

#### Methods in analysis to handle protocol non-adherence and any statistical methods to handle missing data {20c}

Handling of missing data will be performed with multiple imputation. Namely, missing values will be replaced with a set of plausible values containing the natural variability and uncertainty of the right values.

#### Plans to give access to the full protocol, participant-level data and statistical code {31c}

The study protocol and data analysis will be available from the corresponding author upon request.

### Oversight and monitoring

#### Composition of the coordinating centre and trial steering committee {5d}

The coordinating centre is the NIBS unit at IRCCS Santa Lucia Foundation of Rome, Italy. NIBS unit is a specialised division of the laboratory of experimental neuropsychophysiology. The unit, directed from Dr. Koch, is composed of a multidisciplinary team formed by neurologists, neuropsychologists, lab technicians and physiotherapists. The research activity of the NIBS unit aims to understand the mechanisms underlying the plasticity and cortical connectivity of the human brain to develop new therapeutic approaches for the recovery of neurological diseases and enhance clinical practice. The trial steering committee consists of the principal investigator (GK) and co-investigators (SB, GM, GV) who are responsible for supervision, conduction and execution of the research protocol and will issue recommendations for early termination, modifications or continuation of the trial, if necessary. All members of the trial steering committee are experts in the fields of neuroscience and clinical rehabilitation.

#### Composition of the data monitoring committee, its role and reporting structure {21a}

Data monitoring committee is not formally planned for this study for the low-risk factors. The PI is responsible to provide the clinical trials centre (CTC) with a safety annual report, including data about the current recruitment status as well as information about eventual occurring major complications and adverse events. The CTC is an independent internal research board of IRCCS Santa Lucia Foundation of Rome.

#### Adverse event reporting and harms {22}

Expected harms concern exceptional adverse events (AEs) due to TMS that are reported in the literature: (1) skin burn and (2) seizures (of note, seizures have been reported using repetitive TMS in about 10 human subjects, out of tens of thousands tested all over the world), and minor AEs due to TMS or RAT reported in the literature: (1) transient headache, (2) transient dizziness, (3) transient nausea, (4) phosphene-like visual phenomenon if the stimulation will be switched on or off rapidly, (5) paretic upper limb pain.

In the event of an AE, NIBSU staff will provide first aid and notify the event to the emergency office of the IRCCS Fondazione Santa Lucia in Rome. The office, in case of need, will provide specialised assistance in emergency care and management of the case.

All AEs, expected and unexpected, will be reported immediately and in an annual report to the CTC by the PI. All eventually AEs will be collected and reported in the disseminations.

#### Frequency and plans for auditing trial conduct {23}

The steering committee will perform 6-month audits during the entire duration of the trial. For each audit will be evaluated the congruousness with respect to trial procedures, data management and timescale. Any critical issues will be discussed by the steering committee and communicated to interested co-investigator/s. Additionally, auditing will be performed, without anticipated communication, by the CTC board.

#### Plans for communicating important protocol amendments to relevant parties (e.g. trial participants, ethical committees) {25}

All modifications about protocol and procedures must first be approved, by amendment, by the independent Ethics Committee of the Santa Lucia Foundation Hospital. If approved, the changes will be communicated and reported in the online registration of the protocol. All investigators and participants will be immediately informed about the modifications.

### Dissemination plans {31a}

The results dissemination will be carried out by investigators of the study. The results will be published in peer-reviewed journals and reported in conferences presentations and/or posters. After publication, it could be published via dedicated websites/forums and/or social media. The sponsor does not have any role regarding execution, analyses, interpretation of the data, or in dissemination of results.

## Discussion

The objective of the current study protocol is to test the effects of a PPC-M1 PAS in add-on to 15 consecutive sessions of RAT on motor function of the upper limb in chronic stroke via a RCT with a multimodal investigation (clinical, kinematic and neurophysiological). In line with the aim of the current study protocol, Tang et al. in their meta-analysis reported how using high-frequency TMS on the ipsilateral hemisphere is beneficial for motor function, hand strength and hand dexterity in patients diagnosed with sub-acute stroke [29]. Similarly, a network meta-analysis on non-conventional therapies for motor recovery after a stroke reported the high-frequency repetitive transcranial magnetic stimulation as one of the most effective treatments [30]. Similarly, to our protocol, a very recent study tested the combination of another form of non-invasive brain stimulation (dual-tDCS) combined with robot-assisted therapy in chronic stroke finding a positive effect on motor functions in the subgroup of patients with severe cortico-spinal damage treated with real stimulation with respect to the control [31]. Based on the abovementioned scientific evidence, we expect that the stimulation of the central nervous system by the augmented feedback generated by robot-assisted therapy, during the neuromodulation window induced by PAS, can promote a cortical reorganisation which in turn results in a clinical improvement. Therefore, the combination of the two approaches should generate a boost effect on functional motor recovery of UL.

## Trial status

Participant enrolment began on 19 August 2022. The trial is ongoing; the estimated study completion date is October 2023.

## Data Availability

Further information or data can be required from the corresponding author.

## Abbreviations

AEs: Adverse events
ANOVA: Analysis of variance
BBT: Box and Block Test
CTC: Clinical trial centre
Ctrl: Control group
EEG: Electroencephalography
Exp: Experimental group
FMA-UE: Fugl-Meyer assessment scale for upper extremity
M1: Primary motor cortex
NIBS: Non-invasive brain stimulation
PAS: Paired associative stimulation
PLV: Phase locking value
PPC: Posterior-parietal cortex
RAT: Robot-assisted training
rMT: Resting motor threshold
TEPs: Transcranial evoked potentials
TMS: Transcranial magnetic stimulation
TRSP: TMS-related spectral perturbation
UL: Upper limb
